# Integrating Virtual Learning Into a Primarily Cadaveric-Based Curriculum: Medical Students’ Perspectives, Opinions, and Attitudes

**DOI:** 10.7759/cureus.93424

**Published:** 2025-09-28

**Authors:** Justin Lindsay, Aurelia Incristi, Alexander Hull, Anes Gadun, Sydne Ballengee, Graham Kessler, Barbara Kraszpulska

**Affiliations:** 1 Medicine, Wright State University Boonshoft School of Medicine, Dayton, USA; 2 Medical Education, Wright State University Boonshoft School of Medicine, Dayton, USA

**Keywords:** anatomy education, medical education, medical education technology, technology-enhanced education, virtual anatomy technology

## Abstract

Human anatomy is a crucial component of medical education, traditionally taught through human cadaveric dissection, prosections, and formal lectures. However, challenges such as limited exposure to material, difficulty visualizing structures, and cadaver shortages have prompted the integration of virtual anatomy technologies, such as software applications, web-based educational three-dimensional (3D) platforms, and virtual reality, into curricula. These resources offer advantages by accommodating different learning styles, enabling the analysis of anatomical structures in various planes, and increasing exposure to learning materials. Although research has explored modernizing anatomy education with virtual tools, limited focus has been placed on student opinions of integrating virtual resources. This study aimed to examine how the first-time implementation of virtual resources, including web-based educational 3D platforms, augmented reality, and software applications, affected student learning and their opinions on the further integration of virtual learning. An Institutional Review Board (IRB)-approved survey was conducted with first-year medical students enrolled in an introductory human anatomy course at a single institution. Students completed Likert scale and open-ended response questions regarding their experiences using virtual learning tools. Surveys were distributed to students at the start of the course and after each of the three required examinations. Of the 58 students who consented, 42 responded (72.4%), yielding 65 total responses including duplicates. Among the respondents, 69.2% (n=45) felt virtual resources were more effective compared to traditional methods, and 92.3% (n=60) rated them as slightly to extremely useful. Short-answer responses revealed that students had a positive view of virtual tools and found them helpful for achieving course goals. Students saw potential for the greater integration of virtual learning in anatomical education. Overall, students felt that virtual resources complemented traditional cadaveric dissection, particularly among growing cadaver shortages.

## Introduction

An essential component of the medical education curriculum includes human anatomy, which provides students with the necessary knowledge about the structure and organization of the human body to allow for safe and competent future medical practice [1,2]. Traditionally, human anatomy has been taught using didactic lectures and cadaveric dissection. These methods have been reported to have several challenges, including difficulties visualizing body structures, limited exposure to learning material, and a shortage in cadaver supply, making anatomy widely considered to be one of the most difficult subjects for medical students [1-4]. Furthermore, these challenges have been exacerbated by the COVID-19 pandemic, which led to a drastic decrease in the availability of body donations and in-person anatomy teaching sessions [1,5,6].

To offer potential solutions to these challenges, three-dimensional (3D) and virtual anatomy educational technologies have been introduced to academic settings. These technologies offer potential solutions by allowing flexibility in student learning and the analysis of anatomical structures in all planes [1]. Recent evidence by Ogut et al. suggests that learner characteristics, particularly preferred study approaches, can influence study duration and academic performance, highlighting the value of incorporating flexible educational modalities that adapt to different learning styles [7]. They also increase access to anatomical knowledge regardless of student location, increase exposure to learning materials, and improve knowledge application [1,8]. New software applications (“apps”) specifically enhance anatomy medical education by consolidating anatomical knowledge and presenting it in various learning styles that can be tailored to individual students’ needs and preferences [9]. These applications allow students to not only visualize but also manipulate complex structures [8].

Existing literature focuses on the modernization of anatomy education, citing that combining multimodal resources, such as augmented reality, virtual reality, web-based programs, and tablet-based applications, permits anatomy education to move away from its previous didactic history [3,10,11]. It is important to note, however, that recent literature has emphasized that the success of these technologies depends largely on user experience, particularly the perceived ease of use and perceived usefulness, as shown in the technology acceptance model (TAM). Specifically, Alturkustani et al. revealed that self-efficacy, enjoyment, and low computer anxiety were significant factors that led to students’ positive perceptions and intention to use virtual reality platforms regularly [12]. Students were also more likely to accept immersive technology when they were exposed to it in structured ways, underscoring the need for thoughtful implementation strategies [13]. Fang et al. directly evaluated a haptics-based virtual reality simulator and discovered that force-feedback virtual reality platforms increased anatomical comprehension in medical students and residents, providing tangible evidence that virtual reality is valuable for knowledge acquisition [14]. When taken together, these findings show that multimodal virtual resources can directly enhance anatomy education if they are user-friendly and introduced within a structured framework.

Large-scale survey data from Barut et al. demonstrated that anatomy study preferences differ by gender, academic year, and geographical distribution, emphasizing the need for multimodal curricula that combine cadaveric and interactive digital methods to accommodate a variety of learners [15]. However, there has been little emphasis on student-centered feedback or feelings about using virtual learning tools in their medical school anatomy curriculum. No study to date has tracked changes in student attitudes throughout a course after integrating virtual anatomy resources (VAR). This study aims to bridge the gap in understanding students’ attitudes by examining how the integration of virtual learning into a traditionally cadaver-based medical anatomy course at Wright State University impacts first-year medical students’ perceptions and learning experiences.

## Materials and methods

Existing anatomy course structure

At Wright State University Boonshoft School of Medicine (BSOM), human anatomy is taught in Human Architecture (HA) courses separated into HA1 for first-year medical students and HA2 for second-year medical students. Each Human Architecture course consists of online lectures, cadaver dissection, and peer instruction (PI) sessions to model a flipped classroom. All lecture materials are provided to students two weeks before the course begins. Each PI session has assigned prep material (specific online lectures related to the PI topic). Additional sources, such as self-study imaging materials, are also provided in advance. The dissection experience is limited by the number of medical students and space in the dissection laboratory. The HA1 course is 4.5 weeks long and covers the back, thorax, and upper and lower extremity material.

There are three examinations administered during the HA1 course. The first examination includes the back and upper extremity, the second covers the thorax, and the third is a comprehensive final examination of the entire HA1 material. Each examination consists of two parts: multiple-choice questions (MCQ) and a practical examination in the dissection laboratory.

Survey design and implementation

To assess the effect of the first-time implementation of 3D virtual learning resources on student perspectives and attitudes in human anatomy courses, an Institutional Review Board (IRB)-approved Qualtrics survey was distributed to a cohort of first-year medical students enrolled in their HA1 module. The survey questionnaire was created by the authors. Items were reviewed by subject matter experts to confirm alignment with the intended constructs. Based on expert feedback from the module’s professor, we revised ambiguous items to ensure consistency in interpretation. The survey was sent out electronically via email between August 26, 2024, and October 8, 2024. Informed consent was obtained by administering an approved paper consent form, and the participants could terminate their study participation at any time.

The qualitative survey included Likert scale questions and open-ended responses to gain student perspectives on the impact of virtual learning on their perceived performance (Appendices). Students willing to participate self-identified as electing to use virtual learning tools or declining to use virtual learning tools. Students who decided to use virtual anatomy resources (VAR) could choose what best suited them from a suggested list of resources that included Michigan Blue Link, Texas Tech Gross Anatomy, AnatomyZone, 3D Anatomy Atlas, e-Anatomy, and Complete Anatomy (Appendices). The list of resources was created by students who had previously taken the course in conjunction with the HA course director.

The survey was initially distributed on the first day of the HA1 course to assess students’ feelings toward virtual learning in anatomy, then distributed again after each of the two MCQ and practical examinations, and finally administered after the comprehensive final examination. Duplicate responses were aggregated, as survey respondents could answer anonymously, preventing individual tracking over time. Qualitative responses were analyzed via inductive thematic analysis, looking for themes that arose from the data and subsequently organized into theme tables.

Analysis

Following the completion of survey dissemination, qualitative data from short-answer questions were manually organized and interpreted using inductive thematic analysis as described by Braun and Clarke [16,17]. In addition, five Likert scale questions were asked and analyzed by the five levels chosen for each specific question, as discussed below. The figure artwork was created using Microsoft Excel (Microsoft Corp., Redmond, WA).

Ethical approval

This study was approved by the Wright State University Institutional Review Board (IRB number: 2024-567).

## Results

Of 130 students who were asked to participate, 58 students (44.6%) originally signed the IRB consent form. Of those who signed the consent form, the response rate was 72.4% (n=42). Since there were multiple survey administrations, the total aggregated number of survey responses was 65, including duplicate responses. Duplicate responses were aggregated due to the anonymous nature of the survey.

Prior anatomy experience

Survey respondents were asked to indicate if they had ever taken an anatomy course prior to HA1, the introductory anatomy course at BSOM. Of the 65 responses, 20 students (33.3%) stated that they had no prior experience in an anatomy course; 34 (52.3%) took a basic high school- or undergraduate-level anatomy course. Seven students (10.7%) took anatomy at an advanced graduate level, and four (6.1%) took animal anatomy. These responses indicate that most respondents (63%, n=41) had taken a human anatomy course prior to medical school.

The second survey question asked about students’ experience in using any virtual learning tools before medical school. Of the 65 responses, 23 (35.4%) stated that they do not use them at all, 31 (47.7%) said sometimes, and 11 (16.9%) verified that they use virtual resources often.

Enhanced understanding of anatomical structures

**Figure 1:**
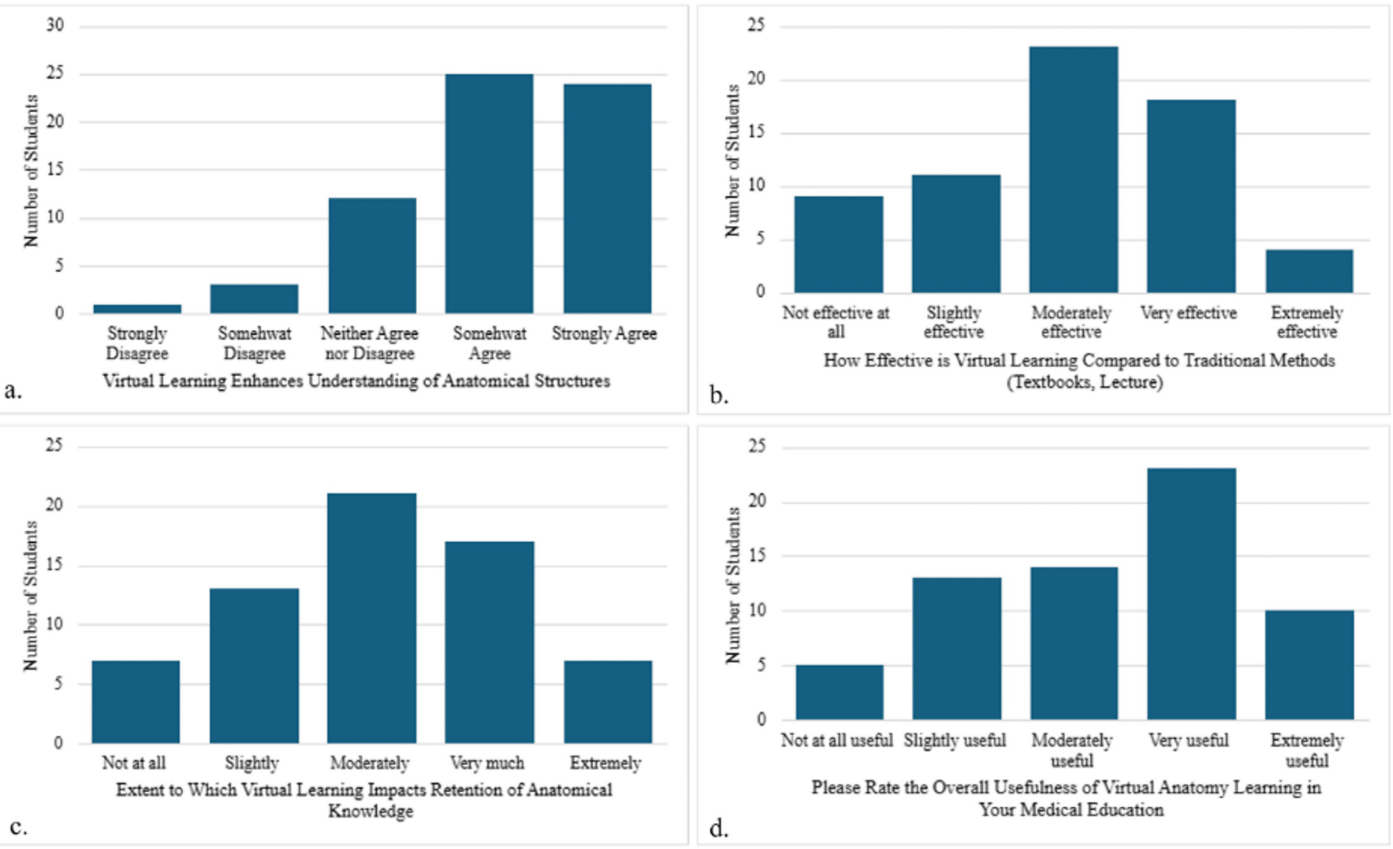
Medical Student Survey Responses to Likert Scale Questionnaire (a) The evaluation of how students felt virtual anatomy resources enhanced their understanding of anatomical structures, (b) the evaluation of how effective students felt virtual resources were compared to traditional learning modalities, (c) the evaluation of the extent to which students felt virtual resources impacted their retention of anatomical knowledge, and (d) student evaluation of the overall usefulness of virtual learning in their medical education

To determine if virtual anatomy learning enhances student understanding of anatomical structures, a Likert scale was used (Figure 1a). The study participants could choose from a range of responses that included “strongly disagree,” “somewhat disagree,” “neither agree nor disagree,” “somewhat agree,” and “strongly agree” with the statement that “virtual anatomy learning enhances my understanding of anatomical structures.” Of the 65 survey responses, 25 students (38.5%) strongly agree, and 24 (36.9%) moderately agree with the statement, meaning that 75.4% (n=49) of the respondents have a positive experience with using virtual anatomy sources and declared that they enhanced their understanding of anatomical structures. Twelve students (18.4%) chose “neither agree nor disagree,” and only four students (6.2%) disagreed with the statement that virtual anatomy learning improves their understanding of anatomical structures.

Effectiveness compared with traditional methods

To further characterize student perception of the impact of virtual resources in introductory human anatomy courses, additional survey questions were provided, and responses were rated using Likert scales. The second survey question asked how effective students found virtual anatomy learning tools compared to traditional methods such as cadaver dissections, online lectures, or textbooks (Figure 1b). The responses included “not effective at all,” “slightly effective,” “moderately effective,” “very effective,” or “extremely effective.” Forty-five students (69.2%) found virtual resources effective compared to traditional learning methods. Only 13.8% of the participants (n=9) declared that the resources were not effective at all, and 17% (n=11) found them slightly effective.

Impact on the retention of anatomical knowledge

When asked to what extent virtual anatomy learning tools improve students’ retention of anatomical knowledge (Figure 1c), student response options included “not at all,” “slightly,” “moderately,” “very much,” or “extremely.” Forty-five students (69.2%) agreed that the resources moderately, very much, or extremely enhanced knowledge retention. Only a small percentage of the participants (10.8%, n=7) disagreed with this statement, and 20% of the students (n=13) felt that the use of virtual resources only slightly improved their retention of anatomy knowledge.

Overall usefulness of VAR in medical education

To assess the overall usefulness of virtual anatomy learning in students’ medical education, a fourth question was provided (Figure 1d). The responses included “not at all useful,” “slightly useful,” “moderately useful,” “very useful,” or “extremely useful.” The results indicated that 92.3% (n=60) of the students found virtual anatomy tools useful. Only five students (7.7%) disagreed.

Perspectives on the impact of virtual anatomy learning in future medical education

When students were asked about the future of virtual anatomy learning evolving in medical education and the potential impact it could have on future generations of medical students, three main themes emerged (Table 1). Students believed that virtual anatomy resources would be useful as a supplement or used in addition to cadaveric learning, with one student commenting that it could “help students visualize structures’ spatial relationships when not physically able to be in the lab.” Students predict the increased usage of virtual anatomy resources as technology is developed and adapted to fit learning needs. Finally, the respondents felt that virtual anatomy resources allowed students to better visualize anatomical structures outside of cadaveric dissections, commenting that virtual anatomy resources could “help institutions with limited funds” for cadavers and be an adequate “substitute…since there are a limited number of donors.”

**Table 1:** Perspectives on the Future of Virtual Anatomy Learning and Its Impact on Medical Education 3D: three-dimensional

Subtheme	Most Common Student Responses
Useful as a supplement/in addition to cadaveric learning	Virtual anatomy resources (VAR) are a good supplement but cannot replace cadaver dissection
	VAR helps visualize structures’ spatial relationships when students are not physically able to be in the dissection laboratory
	VAR is used as a testing method to make sure students can identify structures appropriately
Increased usage as technology begins to develop/be adopted	Medical schools will incorporate virtual anatomy learning. It provides greater ease in seeing deep structures in the human body
	With the continued rapid advance of AI, virtual learning incorporates some of this technology
	VAR will become even more prevalent as technology continues to advance
Allow students to better visualize anatomical structures outside of cadaveric dissections	VAR will improve accessibility
	VAR is good for reinforcing
	VAR helps students see the body in 3D space and be able to imagine where various parts of the body are situated

Students’ perceived limitations to virtual anatomy tools

When asked if students encountered any limitations or challenges while using virtual anatomy learning tools, the main themes that emerged included financial limitations and difficulty with anatomical variations that are not shown in virtual resources (Table 2). Some students indicated that they did not experience any limitations outside of being a novice to the interface, which was overcome with time using the virtual resource. One of the biggest limitations identified was cost, with one student commenting that “paying for so many tools is expensive, especially when you are first trying to figure out what works for you.” Finally, the respondents noted that virtual anatomy resources did not include anatomical variations that could be found on cadaver bodies and that some virtual anatomy resources needed to “[improve] the realistic effect” of their interface.

**Table 2:** Limitations and Challenges of Virtual Anatomy Tools Perceived by Students

Subtheme	Most Common Student Responses
Novice to virtual anatomy with little concern for limitations	Sometimes, the virtual learning resources for anatomy are different from studying a cadaver. However, this is inevitable, and virtual learning should be used to supplement one’s studies in class/dissections
Pricing as a limitation	Pricing and an abundance of different resources to try and choose from are both barriers to receiving adequate virtual education
	The biggest challenge is cost. Paying for so many tools is expensive, especially when you are first trying to figure out what works for you
Difficulty with anatomical variations not provided by virtual learning tools	Variations in some structures that can be seen in patients are not always present in virtual spaces
	It is not always easy to get a full view of the structure digitally. It is also not as easy to get the full picture of all the structures, and it does not prepare students for anatomical variation

Students’ perceived advantages of virtual anatomy tools

Overall, students felt that the main advantage of using VAR compared to traditional resources provided by faculty was the fact that VAR allowed for increased flexibility and availability (Table 3). Students commented that virtual resources allowed them to “study from home” and provided them with a “resource when the professor wasn’t available.”

**Table 3:** Advantages of Virtual Anatomy Tools in Accessibility and Flexibility Compared to Traditional Methods

Subtheme	Most Common Student Responses
Flexibility/accessibility	Accessibility and “ideal” versions of structures are nice. Not being limited to the laboratory for studying helps
	In terms of flexibility and accessibility, virtual anatomy would be very advantageous because students could study from home and out of state, visiting their families
	Virtual anatomy tools provide a more flexible environment for students to effectively learn information. Traditional methods may be more time-intensive and require specific environments (such as dissections)
	They provide a resource when you cannot reach the professor
	Accessible, not time-restricted, and not required to have a cadaver so could be cost-effective

Impact on assessment goals

Lastly, students were asked their perspective on whether using virtual anatomy resources helped them achieve their goal score on assessments (Table 4). An overwhelming theme was that students felt that virtual anatomy learning tools positively impacted their goals. Students commented that virtual resources allowed them “to understand the primary material better” and “interact with spatial relationships when studying at home.” One student also expressed that virtual learning anatomy resources helped them learn origins and insertions of muscles more effectively. Overall, students felt that virtual anatomy learning tools positively impacted their scores on formative assessments.

**Table 4:** Perceived Impact of Virtual Learning Tools on Achieving Goal Assessment Scores VAR: virtual anatomy resources

Subtheme	Most Common Student Responses
Yes, virtual tools impacted my goals in a positive way	VAR helped students to understand the primary material (i.e., lecture and dissection) better, which in turn helped them score well on assessments. They are good supplements rather than primary study tools
	100% helped me interact with spatial relationships when studying at home
	VAR helped especially during review. It has been an excellent introduction and supplement to learning in the laboratory
Did not use virtual tools, but feel that it would have been positive	I did not use virtual tools as much as I would like, but I do believe that it would have helped me increase my scores overall
Virtual tools helped, but other resources helped more	VAR helped, but cadaver and physical exposure helped much more

## Discussion

This study aimed to explore how students felt about the first-time integration of virtual learning tools into a traditionally cadaveric-taught medical school anatomy course and whether students supported the further integration of virtual resources into anatomy curricula. Previous studies have largely ignored student feedback on the modernization of anatomy education, but when considering introducing new modalities of learning into curricula, it becomes imperative to assess the opinions and attitudes of the students impacted by this decision [3,10,11]. Likert scale data concluded that the majority of students felt that virtual resources enhanced their understanding of anatomical structures, were effective when compared to traditional learning methods, enhanced knowledge retention, and were useful. Furthermore, our study revealed that virtual learning plays a significant role as an adjuvant to traditional dissection by allowing for the increased visualization of anatomical structures away from the cadaver laboratory in 3D space. Students had an overall positive view of virtual resource integration and saw the value in the potential further integration of virtual learning into their medical education.

Enhanced understanding of anatomical structures

Our findings demonstrate that the majority of students perceived virtual anatomy resources (VAR) as enhancing their understanding of anatomical structures. These results align with prior research, including a meta-analysis of undergraduate, medical, and residency learners, which reported improved engagement, test scores, and overall satisfaction with virtual anatomy tools [10]. Students in our study particularly valued the ability to tailor their learning to individual weaknesses and to visualize anatomical structures in three dimensions. Furthermore, they determined that the implementation of virtual resources in anatomy education programs has strong indications of enhancing student understanding, immersion, retention, and test performance regarding this complex material [10]. Additionally, it has been demonstrated that virtual reality tools enhanced student spatial comprehension and skill acquisition [14]. The integration of anatomical variation and pathology into VAR further strengthened the perceived educational value [10,14]. By capturing medical students’ attitudes toward integration, our study provides further support that virtual learning is an impactful learning modality.

Effectiveness compared with traditional methods

Students throughout our study considered VAR to be an effective adjunct to traditional modalities such as cadaveric dissection, lectures, and textbooks. In agreement with a study by Korniienko and Barchi, it was found that students in the VR group outperformed those in a textbook group [18]. However, both our findings from medical student opinions and previous research indicate that cadaveric dissection remains essential, with students expressing a desire for continued physical interaction and visualization with anatomical structures [19]. This was primarily reflected when BSOM students were asked if VAR helped them achieve their goal score on assessments. While students overwhelmingly commented that VAR positively impacted their goals, they emphasized that the reasoning for the positive impact was that it helped them understand the primary materials (lectures and dissections) better and was a powerful supplemental tool, but not a full substitute, for reviewing content for examinations [9,19]. Together, these results support the role of VAR as a supplement rather than a replacement for cadaver-based learning [18,19]. The findings of our study suggest that programs with a primary focus on cadaveric learning could see significant benefits from incorporating more modern learning modalities, with increasing support from their students.

Impact on the retention of anatomical knowledge

Our findings provide novel insight into how early exposure can shape learner engagement and perceived benefit, thereby broadening the evidence base for integrating these tools in functioning anatomy education. Many of our own students reported that there was a net positive enhancement among variables such as long-term retention. Students from other studies reported that VAR improved their retention of complex material by allowing the manipulation of structures in three-dimensional space and enabling information to be broken down into more manageable volumes [20,21]. Prior research similarly highlights enhanced immersion and the improved mastery of anatomical relations when students use virtual tools [10]. Nevertheless, some students in our study perceived only modest improvements in retention, underscoring the need for future studies to correlate VAR use with objective performance measures, such as examination scores.

Overall usefulness of VAR in medical education

A large majority of students endorsed VAR as useful for their medical education, citing increased flexibility and accessibility outside of the cadaver laboratory as pertinent positives [18,19]. These findings align with broader trends in anatomy education, where modern learning modalities are increasingly recognized as valuable supplements to traditional instruction [18,19]. Cost, however, was noted by students as a potential barrier to its usefulness, consistent with prior literature [10]. Researchers noted that programs mentioned that the cost of buying the physical VR equipment and the specific software/programs needed for the students was a hindrance to student education [10]. Institutions considering the integration of VAR should weigh these financial challenges carefully, as they may influence equity and access [10]. Furthermore, Barut et al. showed that gender, academic year, and region may cause variation in study preferences, suggesting that VAR adoption will most likely require adaptable approaches to maximize accessibility and effectiveness across diverse populations of learners [15]. Our study also highlights these issues, as students cite the overall usefulness of VAR but are hindered by cost barriers. This insight from our students provides attitudes from students themselves that are significant when considering how to maximize curriculum design.

Advantages and limitations of VAR as perceived by students

The key advantages of VAR identified by students included enhanced three-dimensional visualization, flexibility, and accessibility [20,21]. Limitations included the cost of programs, reduced tactile realism compared to cadaveric dissection, and, in some cases, limited exposure to anatomical variation [10]. Emerging technologies in several anatomy programs that incorporate variation and pathology may help address these gaps [2,22]. Features such as the ability to toggle structures on and off and increase interactivity and immersion appear particularly valuable for learning efficiency [13,14,20,21]. Institutions prioritizing integration should select programs with these capabilities [2,22]. This allows students to synthesize knowledge gained from cadaveric learning with knowledge gained from the 3D virtual space to enhance the understanding and retention of content.

Implementation strategies

A recent study emphasized that virtual learning had the greatest educational impact when these tools were structured within existing curricula [23]. Using specialized study modules in cross-sectional anatomy, Ogut et al. demonstrated that structured virtual modules improved student engagement, proficiency in imaging techniques, and their ability to identify anatomical structures, highlighting the importance of the guided integration of virtual resources rather than stand-alone modules [23]. Einloft et al. demonstrated similar findings that the structured integration of immersive tools significantly increased student acceptance [13]. Our results from current medical students align with this idea, suggesting that VAR is valuable not only in its accessibility but also in its ability to complement and enhance traditional modalities when purposefully embedded into a broader curricular framework.

To maximize the implementation of virtual learning resources, students can use them as pre-dissection study tools to better visualize the anatomy before the cadaver laboratory. Further recommendations would include using the virtual learning tools as flash cards by removing the names of labeled structures and quizzing themselves. This serves as an important consideration for programs, such as ours, looking to integrate VAR into their curriculum, as it plays a significant role in the decision-making process for such a change.

The gradual introduction of VAR through initial voluntary use to assess students’ opinions is an essential step for integration into curricula. Those wishing for further integration can seek to explore the use of assigned modules within virtual learning tools as a next step in implementation. The combination of traditional cadaveric and virtual learning in this study is designed to offer other programs interested in integrating interactive virtual learning modalities a clear understanding of what feedback they can expect from their students.

Limitations and future directions

This study has several limitations. The single-institution design, small sample size, and lack of a randomized control group limit the power of our analysis and the generalizability of the responses from students. Students’ self-identification as electing or declining to use virtual learning tools subjects the study to a self-selection bias, as those more interested in virtual tools may have been more likely to respond. Due to the Family Educational Rights and Privacy Act (FERPA), the authors elected not to pursue protected sensitive material. This limits our ability to draw statistically significant conclusions regarding the impact of virtual learning tools on assessment performance and measure objective outcomes. Future studies seek to correlate students’ attitudes toward virtual learning tools with objective scores on assessments. Additionally, future studies plan to assess which virtual resources were most effective for students and which specific features students found most helpful. Finally, the financial costs associated with credible virtual anatomy learning resources limited student participation. Partnerships between institutions and virtual resource developers may mitigate cost barriers and increase accessibility for students.

## Conclusions

The perspective of medical students on the integration of virtual learning remains underexplored in anatomical education, and our study contributes to addressing this gap by capturing students’ views on the informal use of virtual modalities and where these tools offer the most benefit. Despite being limited to a small sample from a single medical school, our findings provide valuable insight into how students perceive the introduction of new learning tools and what curriculum designers and educators might expect during initial implementation. In the future, medical schools considering virtual learning integration could offer faculty-reviewed resources and assess objective examination outcomes to better understand the role of virtual learning’s impact on curriculum and student success. As this study demonstrates a smaller-scale study, there is significant potential for broader, more formal integration into anatomy curricula, as well as for multi-institutional research to further evaluate student perceptions, academic performance, and the long-term role of virtual learning in medical education.

## Appendices

Survey instrument

Institutional Review Board-approved survey issued to first-year medical students assessing the use of virtual learning in the traditional cadaveric anatomy curriculum

What is your gender?

Male; female; other; prefer not to say

What is your age?

18-22; 22-26; 26-30; 30-34; 34-38; 40-42; 46-50; 50-54; 55+

Have you ever taken an anatomy course prior to Human Architecture 1?

Definitely not; yes, but only at a very basic level (e.g., high school or undergraduate program); yes, at an advanced level (e.g., graduate program); I had animal anatomy but never human anatomy

Have you ever used any virtual learning tools prior to medical school?

Definitely not; sometimes; often; always

When did you or do you anticipate using a virtual learning tool?

At the start of the course; after the first MCQ; after the second MCQ; right before the final; I do not plan on using virtual learning

Please rate your agreement with the following statement: “virtual anatomy learning enhances my understanding of anatomical structures.”

Strongly disagree; somewhat disagree; neither agree nor disagree; somewhat agree; strongly agree

How effective do you find virtual anatomy learning compared to traditional methods (e.g., textbooks and cadavers)?

Not effective at all; slightly effective; moderately effective; very effective; extremely effective

To what extent do you feel virtual anatomy learning improves your retention of anatomical knowledge?

Not at all; slightly; moderately; very much; extremely

Please rate the overall usefulness of virtual anatomy learning in your medical education.

Not at all useful; slightly useful; moderately useful; very useful; extremely useful

How confident are you in applying the knowledge gained from virtual anatomy learning to experiences you may have outside of an anatomy laboratory or lecture?

Not confident at all; slightly confident; moderately confident; very confident; extremely confident

Which virtual resource or application did you use during the course? (Select all that apply)

Michigan Blue Link; Texas Tech Gross Anatomy; AnatomyZone; 3D Anatomy Atlas; e-Anatomy; Complete Anatomy; others

How do you see the future of virtual anatomy learning evolving in medical education, and what potential impact do you think it will have on future generations of medical students? (Please provide 2-3 sentences)

Have you encountered any limitations or challenges while using virtual anatomy learning tools, and if so, how do you think they could be addressed? (Please provide 2-3 sentences)

How effective do you find virtual anatomy learning compared to traditional methods (e.g., lecture textbooks and cadaver dissection)?

What advantages do virtual anatomy learning tools offer in terms of accessibility and flexibility for studying anatomy compared to traditional methods? (Please provide 2-3 sentences)

Do you think that the use of virtual learning tools helped you achieve your goal score on assessments? Why or why not?

List of virtual resources 

The following is the suggested list of well-known virtual anatomy resources provided to a cohort of first-year medical students at a single institution that the students could use to select a virtual anatomy resource: Michigan Blue Link, Texas Tech Gross Anatomy, AnatomyZone, 3D Anatomy Atlas, e-Anatomy, Complete Anatomy, and others (any other virtual resource the student requested to use).

## References

[1] Yang J (2023). Technology-enhanced preclinical medical education (anatomy, histology and occasionally, biochemistry): a practical guide. Adv Exp Med Biol.

[2] Bartoletti-Stella A, Gatta V, Mariani GA (2021). Three-dimensional virtual anatomy as a new approach for medical student’s learning. Int J Environ Res Public Health.

[3] Adnan S, Xiao J (2023). A scoping review on the trends of digital anatomy education. Clin Anat.

[4] Ghosh SK (2017). Cadaveric dissection as an educational tool for anatomical sciences in the 21st century. Anat Sci Educ.

[5] Onigbinde OA, Ajagbe AO, Oyeniran OI, Chia T (2021). Post-COVID-19 pandemic: standard operating procedures for gross anatomy laboratory in the new standard. Morphologie.

[6] Boscolo-Berto R, Tortorella C, Porzionato A, Stecco C, Picardi EE, Macchi V, De Caro R (2021). The additional role of virtual to traditional dissection in teaching anatomy: a randomised controlled trial. Surg Radiol Anat.

[7] Ogut E, Senol Y, Yildirim FB (2017). Do learning styles affect study duration and academic success?. Eur J Anat.

[8] Lewis TL, Burnett B, Tunstall RG, Abrahams PH (2014). Complementing anatomy education using three-dimensional anatomy mobile software applications on tablet computers. Clin Anat.

[9] Zargaran A, Turki MA, Bhaskar J, Spiers HV, Zargaran D (2020). The role of technology in anatomy teaching: striking the right balance. Adv Med Educ Pract.

[10] Miltykh I, Kafarov ES, Covantsev S, Dadashev AS, Skarlis AA, Zenin OK (2023). A new dimension in medical education: virtual reality in anatomy during COVID-19 pandemic. Clin Anat.

[11] Iwanaga J, Loukas M, Dumont AS, Tubbs RS (2021). A review of anatomy education during and after the COVID-19 pandemic: revisiting traditional and modern methods to achieve future innovation. Clin Anat.

[12] Alturkustani S, Durfee A, O'Leary OF, O'Mahony SM, O'Mahony C, Lone M, Factor A (2025). Measuring students' perceptions of virtual reality for learning anatomy using the general extended technology acceptance model for E-learning. Anat Sci Educ.

[13] Einloft J, Bedenbender S, Michelsen M (2024). Structured exposure achieves high acceptance of immersive technology among medical students and educators. Cyberpsychol Behav Soc Netw.

[14] Fang TY, Wang PC, Liu CH, Su MC, Yeh SC (2014). Evaluation of a haptics-based virtual reality temporal bone simulator for anatomy and surgery training. Comput Methods Programs Biomed.

[15] Barut C, Ogut E, Karaer E, Yavuz M (2025). Anatomy study preferences of medical students in relation to gender, academic year and geographical distribution: considerations for anatomy education approaches. Bratisl Med J.

[16] Braun V, Clarke V (2006). Using thematic analysis in psychology. Qual Res Psychol.

[17] Ahmed SK, Mohammed RA, Nashwan AJ, Ibrahim RH, Abdalla AQ, Ameen BM, Khdhir RM (2025). Using thematic analysis in qualitative research. J Med Surg Public Health.

[18] Korniienko IA, Barchi BV (2020). Influence of virtual reality tools on human anatomy learning. Inf Technol Learn Tools.

[19] Alharbi Y, Al-Mansour M, Al-Saffar R, Garman A, Alraddadi A (2020). Three-dimensional virtual reality as an innovative teaching and learning tool for human anatomy courses in medical education: a mixed methods study. Cureus.

[20] Lieu R, Gutierrez A, Shaffer J (2018). Student perceived difficulties in learning organ systems in an undergraduate human anatomy course. HAPS Educ.

[21] Atupele Mwabaleke J, Michael Usman I, Emmanuel Tito A, Edet Obeten K, Umar Isyaku M, Etukudo EM, Fischer VA (2023). Perceptions and challenges faced by undergraduate medical students in studying anatomy: a case study at Kampala International University - Western Campus, Uganda. Adv Med Educ Pract.

[22] Zhang N, Wang H, Huang T, Zhang X, Liao H (2022). A VR environment for human anatomical variation education: modeling, visualization and interaction. IEEE Trans Learn Technol.

[23] Ogut E, Yildirim FB, Senol Y, Senol AU (2025). Comprehensive evaluation of the educational impact and effectiveness of specialized study modules in cross-sectional anatomy: a study on student engagement and learning outcomes. BMC Med Educ.

